# Scope and quality of Cochrane reviews of nutrition interventions: a cross-sectional study

**DOI:** 10.1186/s12937-017-0244-7

**Published:** 2017-04-07

**Authors:** Celeste E. Naude, Solange Durao, Abigail Harper, Jimmy Volmink

**Affiliations:** 1 Centre for Evidence-based Health Care, Faculty of Medicine and Health Sciences, Stellenbosch University, Francie van Zijl Drive, Tygerberg, 7505 South Africa; 2 Cochrane South Africa, South African Medical Research Council, Francie van Zijl Drive, Tygerberg, 7505 South Africa; 3 The Desmond Tutu TB Centre, Faculty of Medicine and Health Sciences, Stellenbosch University, Francie van Zijl Drive, Tygerberg, 7505 South Africa

**Keywords:** Nutrition, Diet, Food, Systematic review, Cochrane

## Abstract

**Background:**

All countries face significant challenges from complex manifestations of malnutrition, which affects one in three people globally. Systematic reviews provide ready-to-use syntheses of quality-appraised evidence to inform decision-making for actions. To enhance the utility and quality of future Cochrane nutrition evidence, we described the scope and quality of all nutrition systematic reviews in the Cochrane Database of Systematic Reviews (CDSR).

**Methods:**

We screened all active CDSR records (31 July 2015) to identify reviews and protocols using pre-specified eligibility criteria and definitions. Duplicate, independent data extraction included criteria for inclusion of studies in completed reviews (PICOS). We assessed methodological quality (AMSTAR), use of GRADE, mapped reviews against 2013 Global Burden of Disease data, and categorised the paradigm (medical, lifestyle and socio-ecological) of the review question. We analysed our results using descriptive statistics.

**Results:**

We screened 8484 records, and included 470 (8%) completed reviews (in 45 Cochrane Review Groups (CRGs)) and 169 (7%) protocols (in 41 CRGs) published by 47 of 53 CRGs with reviews. Most completed reviews were produced by the Pregnancy and Childbirth (*n* = 73), Neonatal (*n* = 64), Metabolic and Endocrine Disorders (*n* = 33), Developmental, Psychosocial and Learning Problems (*n* = 26), Kidney and Transplant (*n* = 18) and Heart (*n* = 18) CRGs. Only 27% (*n* = 129) of reviews had searches for new studies in 2013 or thereafter. Supplementation/supplement interventions were most common (50%; *n* = 235; majority with micronutrients; 73%, *n* = 173), followed by food interventions (20%; *n* = 95). All reviews included randomised controlled trials; about 5% included other designs; 25% used GRADE; the median AMSTAR score ﻿was 9 (interquartile range: 7 t﻿o 10), 51% were high (AMSTAR 9-11) and 49% moderate (AMSTAR 5-8) quality. More than 80% framed questions using a medical paradigm. For top causes of years-of-life-lost, most reviews addressed preterm birth, diabetes and ischaemic heart disease; for leading risk factors for disability-adjusted-life-years, most targeted childhood undernutrition and high body mass index.

**Conclusions:**

Nutrition reviews comprised 8% of active CDSR records, were widely distributed across nearly all CRGs and reflected the double nutrition burden. This analysis presents a comprehensive description of the scope and quality of Cochrane nutrition reviews, and identifies gaps for future activities to support actions to address the nutrition burden, in line with the current nutrition agenda and impetus.

## Background

The nutrition landscape has changed over the past 15 years and most countries are now faced with connected, complex and overlapping nutrition burdens. Multiple manifestations of malnutrition (undernutrition, overnutrition and micronutrient malnutrition) at community, household and individual levels, have become the ‘new normal’ [1]. Dealing with malnutrition, in all its forms, in the same place, at the same time is a problem for nearly half of all countries [2], and every nation faces a significant public health challenge from malnutrition, which affects one in three people globally [1–3].

Finding and implementing effective, scalable and sustainable solutions to address the complex, multi-sectoral nutrition burden is challenging for all stakeholders, particularly since decision-makers often have to deal with diverse and competing interests. Synthesised evidence in which scientifically defensible methods have been used (systematic reviews) can be a valuable tool for translating knowledge to action [4, 5]. Over the past 20 years, Cochrane – a global independent network of about 37 000 contributors from more than 130 countries - has helped transform the way health decisions are made by producing high-quality, relevant, up-to-date systematic reviews published in the Cochrane Library. However, attention has previously been drawn to the lack of nutrition-relevant Cochrane reviews [6, 7]. Furthermore, a recent assessment of Cochrane and non-Cochrane systematic reviews of nine pre-specified interventions from four nutrition areas, reported substantial variability in nutrition-specific methods, and that the scope of some interventions was broad, with poor focus on nutrition-specific indicators [8]. There are issues unique to nutrition topics that should be considered when preparing systematic reviews to aid interpretation of study findings, and enhance overall quality, applicability, and certainty of the evidence. These include background nutrient exposure, baseline nutritional status, multiple biologic functions of nutrients and the variable nature of nutrient interventions [9]. Other challenges in nutrition systematic reviews include lack of true placebo in controlled trials, blinding not always being possible, limitations of different methods or instruments used for assessing dietary intakes, inadequate duration of follow-up to observe a measurable effect over the study duration, as well as poor reporting or ill-defined diets, making it problematic to convincingly attribute an effect to a well-defined treatment compared to a well-defined control. Furthermore, there are the methodological challenges related to evaluating nutrition interventions implemented through sectors other than health, such as agriculture, social protection and trade.

Nutrition is a risk factor for most other dominant health burdens including infectious diseases, maternal and perinatal conditions and non-communicable diseases, especially in low and middle income countries. Nutrition is also an outcome of development processes in society and often connected to issues such as education, care, sanitation and hygiene, women’s empowerment and economic growth, and can therefore be viewed as both an input to and an outcome of sustainable development [10]. Various approaches have been used to conceptualize, describe and address nutritional challenges in society. The multiple interacting factors at many levels can create significant complexity. There may be different intervention components, non-linear pathways and feedback loops between the intervention and outcomes, and interactions between indirect and direct effects of an intervention [11]. Interventions themselves may be simple (e.g. a single component targeting an individual level outcome with a linear pathway) or complex (e.g. systems, components or processes targeting multiple health and non-health outcomes at community level). [11].

**Fig. 1:**
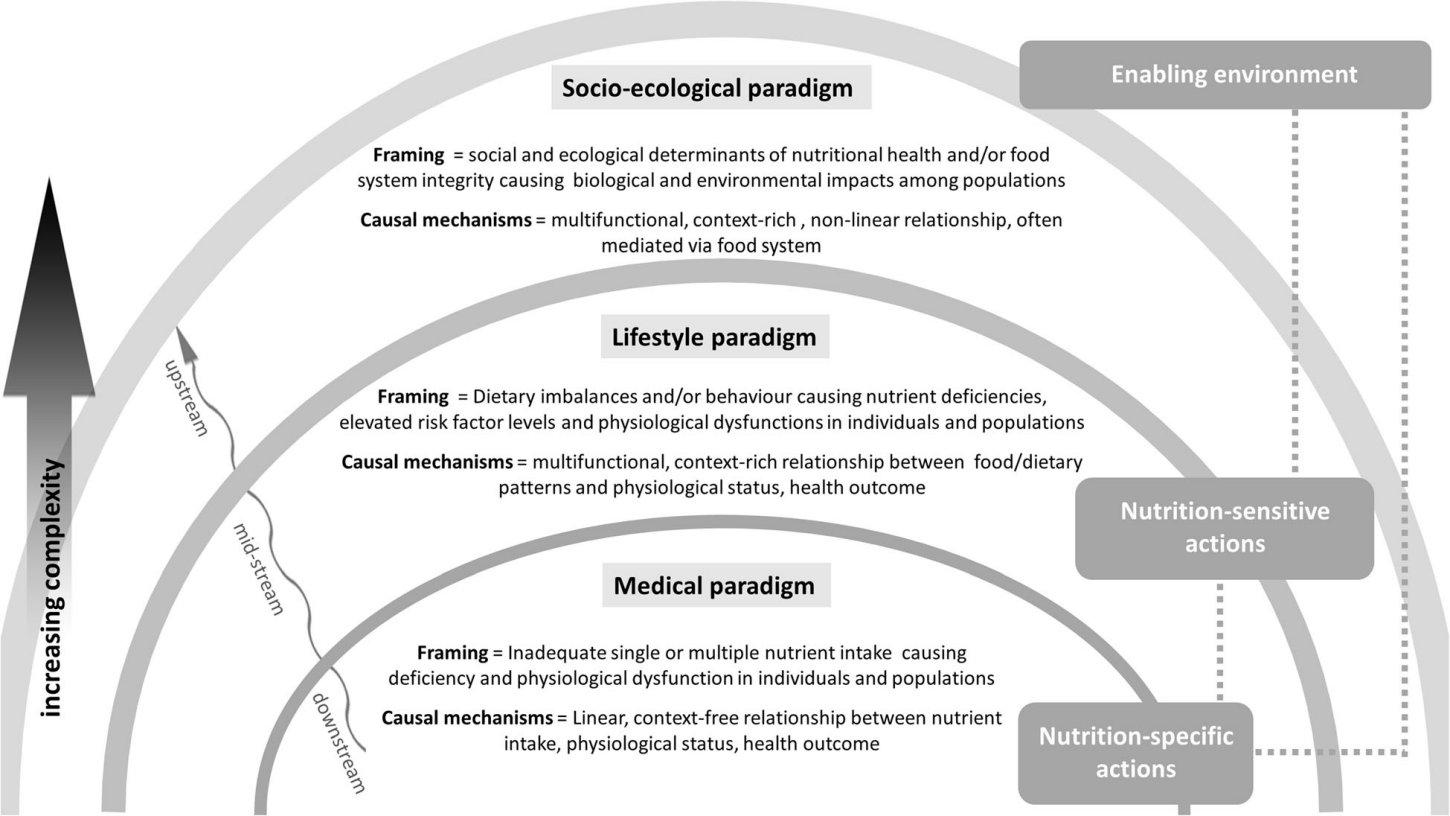
Graphic representation of the three paradigms (medical, lifestyle and socio-ecological) that conceptualize how the relationship between food and health is viewed and how the causes of nutrition problems are framed [12], along with the broad categories of nutrition interventions (nutrition-specific, nutrition-sensitive) and the enabling environment for nutrition improvement to support these interventions [2, 13]

Since approaches to assessing interventions are closely linked to putative causal pathways, the perspectives adopted by researchers play an important role in evaluating cause-effect relationships. Lawrence has identified three paradigms (medical, lifestyle and socio-ecological) within which the relationship between food and health can be viewed, and which specifically influence how the causes of nutrition problems are framed [12]. The medical paradigm incorporates a linear view of single or multiple nutrient deficiencies and their impacts on health, without considering context or other components or factors affecting these relationships. The lifestyle paradigm goes wider, considering multi-functional, non-linear relationships between food, dietary patterns and other factors, such as behaviour. Broadest of all is the socio-ecological paradigm, which views the relationships between nutrition and health within social and ecological settings, with consideration of food and other relevant systems. We have graphically represented these three paradigms in Fig. 1, and overlaid the broad categories of actions that are likely relevant to each paradigm, namely nutrition- specific interventions, nutrition-sensitive interventions and an enabling environment for nutrition improvement [2, 13]. Nutrition-specific interventions address the immediate causes of undernutrition, such as inadequate dietary intake, and some of the underlying causes like feeding practices and access to food. Nutrition-sensitive interventions would address some of the underlying and basic causes of malnutrition by incorporating nutrition goals and actions throughout a wide range of sectors. They can also serve as delivery platforms for nutrition-specific interventions [13].

In order to enhance the utility and quality of future Cochrane nutrition evidence and inform the work of the proposed Cochrane Nutrition Field (subsequently established in August 2016), we conducted an overview of the scope and quality of all nutrition-related systematic reviews published in the Cochrane Database of Systematic Reviews (CDSR). Our objectives were to (1) screen all completed and published reviews and review protocols (excluding withdrawn records) published in the CDSR to determine the number and percentage of nutrition-related reviews completed and in progress; (2) determine the criteria for inclusion of studies in completed reviews; (3) assess the methodological quality of completed reviews and document the use of the Grades of Recommendation, Assessment, Development and Evaluation (GRADE) system [14]; and (4) categorise and describe the completed reviews in relation to the global burden of disease [15, 16] and the paradigm (medical, lifestyle and socio-ecological) of the review question [12].

## Methods

### Selection of reviews

Pairs of authors (CN, SD, AH and JV), using pre-specified eligibility criteria, independently screened all active titles and abstracts, and full-texts if necessary, in the CDSR up to 31 July 2015, to identify nutrition-related reviews and protocols. Nutrition reviews were defined as those that investigated the effectiveness of (1) diets and dietary patterns; foods; formulated, fortified or enriched foods or nutritional products and nutrients and bioactive non-nutrients naturally in foods, delivered orally, enterally or parenterally; (2) nutrition education, promotion, counselling, and programmes; coordination of care or delivery of foods or nutrients; and (3) any policies, programmes or systems which aimed to influence outcomes clearly distinguishable as nutrition-related. We included reviews and protocols that evaluated plant or other components used in food (e.g. cinnamon) and nutrition-related interventions combined with other types of interventions (e.g. diet and exercise). However, we excluded reviews or protocols that assessed pharmaceutical or herbal medicines and products only, those that focused on components of plant origin not used in food (e.g. Echinacea) and where diet or nutrition was not explicit in the title or abstract. Disagreements were resolved through discussion among the authors.

### Data extraction and management

We obtained the full texts of all included reviews and protocols and two authors independently extracted the following information for entry into a piloted, standardised data extraction form [17]: unique Cochrane ID number, title, CRG that produced the protocol or review, and year of publication as stated next to the phrase “Publication status and date” in each full-text. For completed reviews, we also extracted year assessed as up-to-date by capturing the year specified next to the phrase “Review content assessed as up-to-date”. A Cochrane review should usually be updated about every two years and updating must involve a search for new studies, which if identified must be assessed for inclusion and if eligible, incorporated into the review [18]. The date at which the review content is assessed as up-to-date refers to the date of the most recent search for new studies. Other extracted domains were: use of the GRADE system, inclusion of a summary of findings table in the review [14], and pre-specified criteria for selecting studies for inclusion in the review, i.e. types of participants, interventions, comparators, outcomes and study designs (PICOS). We categorised and described the extracted PICOS information as shown in Table 1. Interventions in completed reviews were classified as either a primary nutrition intervention or as a nutrition intervention delivered in combination with, or as part of, other interventions.

**Table 1:** Categorisation of types of participants, interventions, comparators, outcomes and study types

Data domain	Categories used for data extraction
Participants	• pregnant women • mothers and infant pairs • infants • children of preschool-aged children • school-aged children • adults • elderly • postmenopausal women • participants with a condition(s) or not
Interventions	• foods (e.g. whole foods, food products, complete diets or dietary patterns, specially formulated foods, complete nutritional formulas, breastfeeding) • supplementation/supplements (e.g. single or multiple nutrients, bioactive non-nutrients, plant components) • combined food and supplementation/supplements • nutrition education, counselling and coordination of care • policies, programmes or systems that influence nutrition-related or nutrition-sensitive outcomes • other, if no component of the intervention could be categorised as any of the above
Comparator	• placebo • no intervention • usual care • different intervention • other
Outcomes	• mortality • clinical or nutritional status assessments (e.g. anthropometry, clinical and biochemical measurements) • frequency and/or severity of disease • diet quality and/or variety • food/nutrient/dietary intake • diet-related behaviours (including eating behaviour) • other non-diet-related behaviours • withdrawal from the study, drop-out or adherence-related • adverse events, side-effects and/or safety • cost-effectiveness or economic • quality of life • other
Study designs	• randomised controlled trials (including parallel or cross-over design); • experimental non-randomised studies (non-randomised controlled trials, controlled before-after studies, interrupted time series and repeated measures studies) • observational studies (cohort, case-control, cross-sectional)

We mapped the topics addressed by all completed reviews against the top 50 causes of global years of life lost (YLLs) in 2013 [16] and the 25 leading level three global risk factors for disability adjusted life years (DALYs) [15], according to the 2013 Global Burden of Disease Study. We also assessed the scope of reviews by categorising them into three paradigms: medical, lifestyle and socio-ecological [12] (Fig. 1).

Two authors independently assessed the methodological quality of all completed reviews that included at least one primary study using the validated AMSTAR tool [19] and resolved discrepancies by discussion. One point was awarded for each of the domains judged as meeting the recommended criterion, and points were summed to calculate a total score for each review. Total scores of 0 to 4 indicated low methodological quality, 5–8 moderate quality, and 9–11 high quality [20].

### Analysis

We analysed the data using Stata/IC for Windows [21], and used descriptive statistics to explore and present the results. Tables, charts and descriptive summaries present the number of included reviews and protocols within each CRG, as well as data on participants, interventions/exposures, comparators, outcomes, study designs, and methodological quality.

## Results

**Fig. 2:**
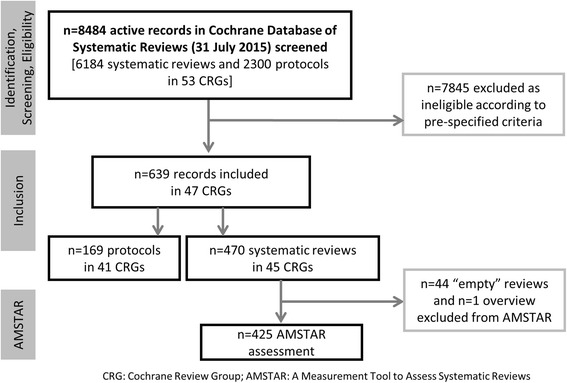
Flowchart illustrating the search results and selection process of nutrition reviews and protocols in the Cochrane Database of Systematic Reviews, and methodological quality assessment of systematic reviews [19]

We screened 8484 active records in the CDSR at 31 July 2015 and included a total of 470 completed systematic reviews (in 45 CRGs) and 169 protocols for systematic reviews (in 41 CRGs) (Fig. 2). One of the included reviews was an overview of systematic reviews, and we extracted data for all applicable fields from the overview. Of the 470 reviews, 425 included at least one primary study and we assessed the methodological quality of these reviews using AMSTAR. We excluded from the AMSTAR assessment 44 ‘empty’ reviews that contained no eligible studies, and the overview, as the tool is applicable to systematic reviews.

### Nutrition reviews by Cochrane Review Groups

**Fig. 3:**
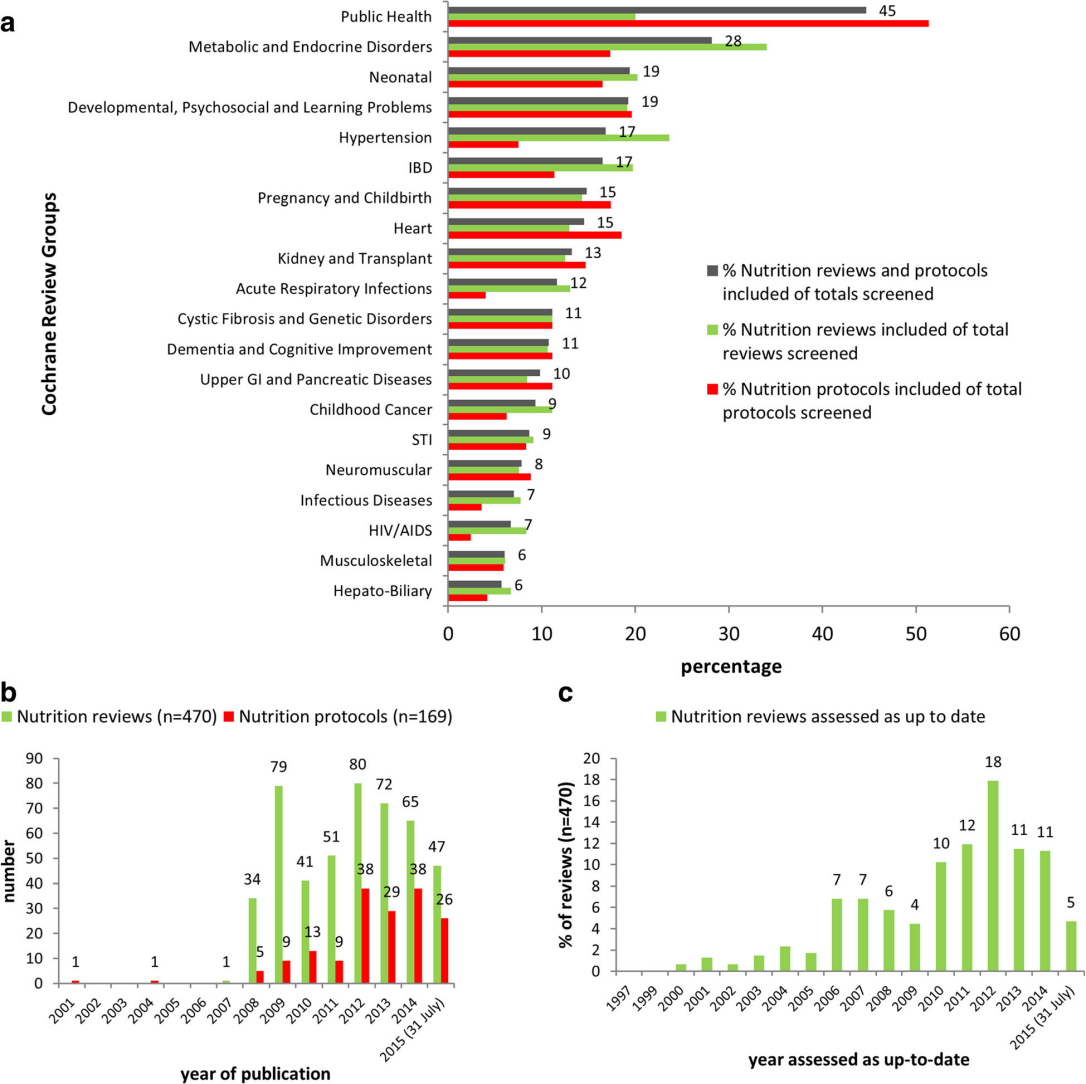
**a)** Proportions of nutrition reviews and protocols included of the total reviews and protocols screened in Cochrane Review Groups where 6% or more of all records screened per Group were included (numbers next to bars indicate the percentages of both reviews and protocols); **b**) Number of nutrition reviews and protocols published in the Cochrane Database per year; **c**) Percentage of nutrition reviews (*n* = 470) assessed as up-to-date by year

Table 2 details the number of records (reviews and protocols) screened and included per CRG in descending order of number of records included. Of the 470 completed nutrition reviews included, most were produced by the Pregnancy and Childbirth (*n* = 73), Neonatal (*n* = 64), Metabolic and Endocrine Disorders (*n* = 33), Developmental, Psychosocial and Learning Problems (*n* = 26), Kidney and Transplant (*n* = 18), and Heart (*n* = 18) groups (Table 2). The greatest numbers of included protocols (reviews in progress) were produced by the Public Health (*n* = 19), Pregnancy and Childbirth (*n* = 16), Neonatal (*n* = 15), Developmental, Psychosocial and Learning Problems (*n* = 11), Kidney and Transplant (*n* = 10), and Heart (*n* = 10) groups (Table 2). As a proportion of total active records produced by each CRG at the date of screening, the Public Health (45%), Metabolic and Endocrine Disorders (28%), Neonatal (19%), Developmental, Psychosocial and Learning Problems (19%) groups had the most nutrition reviews and protocols (Fig. 3a). However, groups with the highest proportion of completed nutrition reviews were the Metabolic and Endocrine Disorders (34%) and Hypertension (24%) groups, and those most nutrition protocols were Public Health (51%) and Developmental, Psychosocial and Learning Problems (20%) groups (Fig. 3a).

**Table 2:** Number of records (reviews and protocols) screened and included per Cochrane Review Group in descending order by number of records included

Cochrane Review Group (CRG)	Number of records screened	Number of reviews included	Number of protocols included
Pregnancy and Childbirth	602	73	16
Neonatal	407	64	15
Metabolic and Endocrine Disorders	149	33	9
Developmental, Psychosocial and Learning Problems	192	26	11
Heart	193	18	10
Kidney and Transplant	212	18	10
Public Health	47	2	19
Acute Respiratory Infections	163	18	1
Cystic Fibrosis and Genetic Disorders	171	16	3
Inflammatory Bowel Diseases	115	14	5
Dementia and Cognitive Improvement	167	13	5
Hepato-Biliary	299	12	5
Musculoskeletal	281	11	6
Hypertension	95	13	3
Airways	346	12	3
Neuromuscular	153	9	3
Upper GI and Pancreatic Diseases	122	5	7
Infectious Diseases	157	10	1
HIV/AIDS	149	9	1
Colorectal Cancer	179	5	4
Injuries	177	8	1
Oral Health	205	7	2
Pain, Palliative and Supportive Care	242	8	1
Wounds	165	6	2
Common Mental Disorders	213	5	2
Gynaecology and Fertility	236	7	0
Schizophrenia	268	4	3
Vascular	178	5	2
Anaesthesia, Critical and Emergency Care Group	244	4	2
Eyes and Vision	196	5	1
Gynaecological, Neuro-oncology and Orphan Cancer	198	4	2
Bone, Joint and Muscle Trauma	146	2	2
Childhood Cancer	43	3	1
Stroke	212	2	2
Ear, Nose and Throat	141	1	2
Effective Practice and Organisation of Care	162	2	1
Epilepsy	101	2	1
Multiple Sclerosis and Rare Diseases of the CNS	61	3	0
Skin	114	3	0
Consumers and Communication	79	2	0
Movement Disorders	92	2	0
Sexually Transmitted Infections	23	1	1
Tobacco Addiction	82	1	1
Urology	52	1	1
Drugs and Alcohol	97	1	0
Haematological Malignancies	99	0	1
Incontinence	94	0	1
Back and Neck	87	0	0
Breast Cancer	76	0	0
Fertility Regulation	87	0	0
Lung Cancer	38	0	0
Methodology Review	39	0	0
Work	38	0	0
Totals	8484	470	169

### Year of publication and up-to-date status of completed reviews

The first Cochrane nutrition review was published in 2007, with 44% (*n* = 206) of reviews being published up to and including 2011, and the rest from 2012 to 31 July 2015 (56%; *n* = 264) (Fig. 3b). About a fifth (21%; *n* = 36) of nutrition protocols were published from 2008–2011, with the majority being produced from 2012 onwards (78%; *n* = 131) (Fig. 3b). Notably, the first nutrition protocol published in 2001 and the second published in 2004 have yet to be published as completed reviews. About 70% (*n* = 341) of completed reviews had their review content assessed as up-to-date prior to 2013 (Fig. 3c), thus their most recent searches for new studies were done prior to 2013. The remaining reviews had more recent searches for new studies (2013 to 31 July 2015).

### Description of included reviews

#### Types of participants

Two third of reviews assessed interventions for participants with a specified condition(s) (67%; *n* = 316) and the remaining third (33%; *n* = 154) evaluated interventions for preventing or reducing risk for a specified condition(s) (Table 3). About a third of reviews did not define eligible participants by age or life-stage (32%; *n* = 151). Of reviews that pre-specified eligible participants by age or life-stage category, most included adults (22%; *n* = 104), followed by infants (14%; *n* = 66), pregnant women (11%; *n* = 53) and infants, preschool- and school-aged children (7%; *n* = 31). The remaining reviews included participants in other life stage categories (Table 3).

**Table 3:** Total number of included reviews by pre-defined participant categories, and stratified according to whether the review assessed treatment or prevention of a condition(s)

Participant categories (in descending order of total number)	Total nutrition reviews (*n* = 470)	Reviews assessing treatment (*n* = 316; 67%)	Reviews assessing prevention or risk reduction (*n* = 154; 33%)
No age group specified	151	131	20
Adults	104	73	31
Infants (<1year)	66	50	16
Pregnancy	53	12	41
Infants, preschool- and school-aged children	31	21	10
Mother and infant pairs	17	2	15
Infants and preschool-aged children	15	5	10
Older people (>65 years)	8	4	4
Preschool- and school-aged children	8	8	0
School-aged children	6	2	4
Preschool- and school-aged children and adults	4	4	0
Postmenopausal females	3	2	1
School-aged children and adults	3	2	1
Mother and infant pairs and preschool-aged children	1	0	1
Preschool-aged children	0	0	0

#### Types of interventions and comparators

**Fig. 4:**
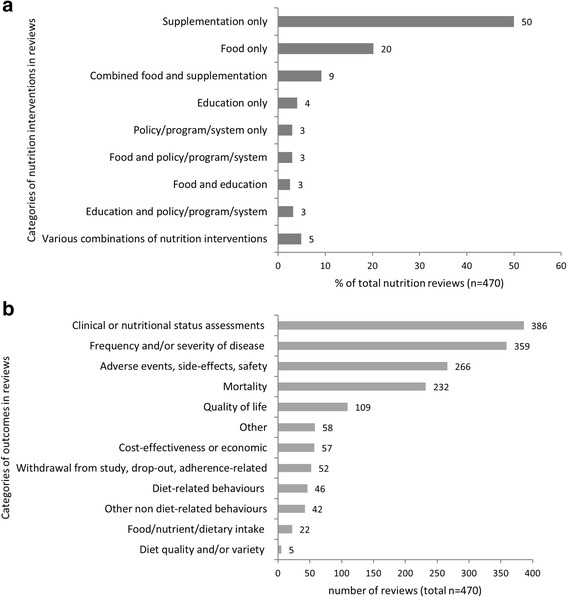
**a** The percentage of completed nutrition reviews (*n* = 470) that examined the various categories of nutrition interventions **b**) The numbers of completed nutrition reviews that included the various categories of outcomes

Nutrition was the primary intervention in most included reviews (86%; *n* = 406), and was delivered in combination with or as part of other interventions in the remaining reviews (14%; *n* = 64). Reviews of nutritional supplementation/supplements were the most common (50%; *n* = 235), followed by food interventions (20%; *n* = 95), combined food and supplementation interventions (9%; *n* = 43) and nutrition education (4%; *n* = 19) (Fig. 4a). Policy/program/system interventions comprised 3% of reviews (*n* = 14). Combinations of food and policy/program/system interventions, food and education, and education and policy/program/system interventions each made up about 3% of reviews. The small proportion of reviews (5%) included different combinations of the specified intervention categories (Fig. 4a). Table 4 provides examples of reviews by type of intervention category.

**Table 4:** Examples of review titles for the main intervention categories

Supplementation/supplement interventions:	
micronutrients	Micronutrient supplementation in children and adults with HIV infection Daily oral iron supplementation during pregnancy Vitamin D compounds for people with chronic kidney disease not requiring dialysis
alternative supplements	Probiotics for preventing acute upper respiratory tract infections Garlic for the common cold Glucosamine therapy for treating osteoarthritis
Food interventions:	
diets	Ketogenic diet and other dietary treatments for epilepsy ’Mediterranean’ dietary pattern for the primary prevention of cardiovascular disease Baby-led compared with scheduled (or mixed) breastfeeding for successful breastfeeding
complete formulas	Enteral nutritional therapy for induction of remission in Crohn’s disease Soy formula for prevention of allergy and food intolerance in infants Enteral versus parenteral nutrition for acute pancreatitis
single foods	Effect of cocoa on blood pressure Green tea (Camellia sinensis) for the prevention of cancer Honey for acute cough in children
food groups	Increased consumption of fruit and vegetables for the primary prevention of cardiovascular diseases Whole grain foods for the prevention of type 2 diabetes mellitus Wholegrain cereals for coronary heart disease
Combined food and supplementation interventions	
	Interventions for the prevention of nutritional rickets in term born children Dietary interventions for multiple sclerosis Omega 3 fatty acids for preventing or slowing the progression of age-related macular degeneration
Nutrition education interventions	
	Dietary advice for treatment of type 2 diabetes mellitus in adults Antenatal breastfeeding education for increasing breastfeeding duration Dietary advice given by a dietitian versus other health professional or self-help resources to reduce blood cholesterol

Further scrutiny of the supplementation reviews revealed that nearly three quarters (73%; *n* = 173) evaluated micronutrients (e.g. vitamins, minerals, trace elements), while others assessed alternative supplements, such as amino acid derivatives (e.g. carnitine), probiotics, food and spice extracts (e.g. garlic) and phytochemicals (e.g. flavonoids) (20%; *n* = 47) or combinations of these (7%) (e.g. antioxidant supplements including conventional micronutrients and phytochemical compounds).

With regards to food interventions, half of the reviews studied the effects of whole diets (50%; *n* = 66), nearly a third (31%; *n* = 41) investigated complete formulas (e.g. enteral nutrition, infant formulas, ready-to-use therapeutic foods), 8% (*n* = 11) single foods (e.g. sweet potato, honey, fermented milk), 4% (*n* = 5) food groups (e.g. wholegrain cereals), and the rest studied combinations of these.

Most reviews pre-specified more than one type of comparator. The most common comparator was placebo (63%; *n* = 297), followed by no intervention (51%; *n* = 238), a different intervention (51%; *n* = 237), or usual care (25%; *n* = 119). While these three were also the most common comparators in supplementation reviews, usual care was most commonly used as the comparator in reviews of food interventions.

#### Types of outcomes

Reviews most frequently assessed the following pre-specified outcomes: clinical or nutritional status assessments (82%; *n* = 386), frequency/severity of disease (76%; *n* = 359), adverse events, side-effects, and safety (57%; *n* = 266), and quality of life (23%; *n* = 109) (Fig. 4b). Fewer reviews included outcomes on economics or cost-effectiveness (12%); withdrawal, drop-out or adherence (11%); and diet-related behaviours (10%) (Fig. 4b). Less than 10% of reviews examined the effects of nutrition interventions on non-diet-related behaviours, food/nutrient/dietary intake, and dietary quality or variety (Fig. 4b).

#### Study designs

All nutrition reviews included randomised controlled trials (RCTs) and 3% (*n* = 16) included RCTs and experimental non-randomised studies. Very few reviews included RCTs and observational studies (2%; *n* = 8). Six reviews (1%) included RCTs, experimental non-randomised and observational studies. The single overview included only systematic reviews.

### Methodological quality on included reviews and use of GRADE

**Fig. 5:**
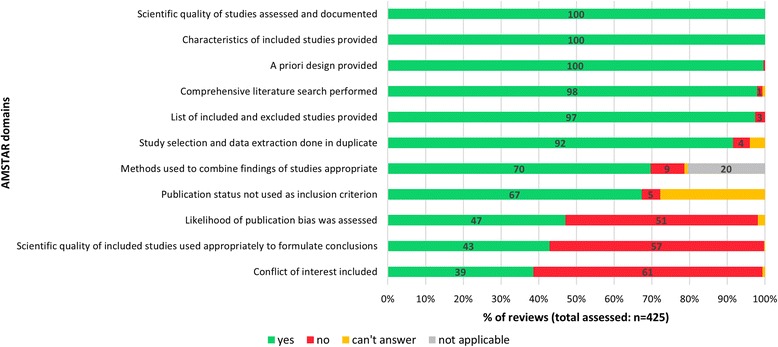
Percentages of reviews judged as meeting the recommended criterion specified for each of the 11 AMSTAR domains (*n* = 425 reviews methodological quality assessed using AMSTAR tool)

The median AMSTAR score of the 425 eligible reviews assessed was 9 (interquartile range: 7–10), indicating high methodological quality. Half of the reviews were judged to be of high quality (51%; *n* = 218) (AMSTAR score 9–11), and the other half (49%; *n* = 207) were considered to be of moderate quality (AMSTAR score 5–8). AMSTAR domains most frequently judged to have shortcomings in the reviews assessed were those related to the appropriate use of the scientific quality of included studies in formulating conclusions (57%), the assessment of the likelihood of publication bias (51%), and the assessment of potential conflicts of interest in the primary studies included in the reviews (61%) (Fig. 5). All reviews declared their sources of support (*n* = 470). A quarter of nutrition reviews (25%; *n* = 118) used the GRADE system for rating quality of evidence and a Summary of Findings table for presenting results.

### Reviews addressing the burden of disease and paradigms

Forty-two percent (*n* = 196) of nutrition reviews addressed one or more of 28 of the top 50 causes of global YLLs in 2013 [16] related to nutrition (Table 5). The remaining 22 causes are not known to have any direct nutritional link (e.g. road injuries, drowning, and syphilis). The YLL causes addressed by the greatest number of reviews were preterm birth (21%; *n* = 42), ranked as the 7^th^ leading cause of YLLs, diabetes (10%; *n* = 26), ranked 17^th^. Ischaemic heart disease (rank 1), maternal disorders (rank 26), asthma (rank 32), diarrhoeal diseases (rank 4), and HIV/AIDS (rank 6) were addressed by between 5 and 10% of the 196 reviews (Table 5).

**Table 5:** Mapping of number of reviews addressing top 50 causes of global years of life lost in 2013 [16] and the top 25 global risk factors for disability adjusted life years (both sexes combined) [15], according to the 2013 Global Burden of Disease Study

Rank	Top 50 causes of global years of life lost (YLLS) in 2013	Number of nutrition reviews (total: *n* = 196)	Rank	Top 25 global risk factors for disability adjusted life years (DALYs) in 2013	Number of nutrition reviews (total: *n* = 176)
7	Preterm birth	42	4	Childhood undernutrition	48
17	Diabetes	26	3	High body mass index	27
1	Ischaemic heart disease	14	24	Low omega-3	24
26	Maternal disorders	11	19	Suboptimal breastfeeding	18
32	Asthma	11	18	Iron deficiency	15
-	(unspecified cancer – not ranked)	10	1	High blood pressure	15
4	Diarrhoeal diseases	10	11	High sodium	8
6	HIV/AIDS	9	13	High total cholesterol	6
19	Chronic kidney disease	8	6	Alcohol use	3
2	Lower respiratory infections	7	5	High fasting plasma glucose	3
33	other cardiovascular	7	10	Low fruit	2
45	Iron deficiency anaemia	6	15	Low whole grains	2
18	Protein-energy malnutrition	6	15	Low vegetables	2
24	Hypertensive heart disease	4	8	Unsafe water	1
27	Colorectal cancer	4	17	Low physical activity	1
3	Cerebrovascular disease	3	25	Low fibre	1
34	Fire and heat	3	No nutrition reviews for [Rank]: [2] Smoking, [7] Household air pollution, [9] Unsafe sex, [12] Ambient particulate matter, [14] Low glomerular filtration rate, [16] unsafe sanitation, [21] handwashing, [22] drug use, [23] low nuts and seeds		
43	Measles	3			
47	Brain cancer	2			
49	Endocrine, metabolic, blood & immune disorder	2			
8	Malaria	1			
10	Congenital anomalies	1			
11	Tuberculosis	1			
12	Chronic obstructive pulmonary disease	1			
16	Neonatal sepsis	1			
25	Stomach cancer	1			
36	Sickle cell	1			
39	Leukaemia	1			
No nutrition reviews for [Rank]: [5] Road injuries, [9] Neonatal encephalopathy, [13] Cirrhosis, [14] Self harm, [15] Lung cancer, [20] Drowning, [21] Liver cancer, [22] Interpersonal violence, [23] Meningitis, [28] Falls, [29] Alzeimer’s, [30] Breast cancer, [31] cardiomyopathy, [35] syphilis, [37] typhoid fever, [38] oesophageal cancer, [40] interstitial lung disease, [41] rheumatic heart disease, [42] peptic ulcer disease, [44] pancreatic cancer, [46] cervical cancer, [48] pulmonary aspiration, [50] lymphoma					

Sixteen of the leading 25 risk factors for disability adjusted life years (DALYs) were addressed by 37% of reviews (*n* = 176). Some reviews addressed more than one risk factor. All except one of the remaining nine risk factors have no direct nutritional link (Table 5). The risk factor addressed by the greatest number of reviews was childhood undernutrition (27%; *n* = 48; rank 4), followed by high body mass index (15%; *n* = 27; rank 3), and low omega-3 fatty acid intake (14%; *n* = 24; rank 24). Suboptimal breastfeeding (rank 19), iron deficiency (rank 18), high blood pressure (rank 1), and high sodium intake (rank 11) were addressed by between 5 and 10% of the 176 reviews (Table 5).

The majority of included reviews (82%; *n* = 386) framed their questions using a medical paradigm, in which inadequate intake of single or multiple nutrients causes deficiency and physiological dysfunction in individuals and populations, with a linear, context-free relationship between nutrient intake, physiological status, and health outcomes (Fig. 1) [12]. Nearly one in five reviews (17%; *n* = 78) framed questions using the lifestyle paradigm, where behaviours or dietary imbalances cause nutrient deficiencies, elevated risk factor levels and physiological dysfunctions in individuals and populations, with a multifunctional, context-rich relationship between food/dietary patterns, physiological status, and health outcomes [12]. The socio-ecological paradigm, which incorporates social and ecological determinants of nutritional health and food system integrity, was used by only six reviews (1%).

## Discussion

### Summary of main findings

Similar to previous reports [8], we found that the methodological quality of Cochrane nutrition reviews was high (AMSTAR) [19]. Many reviews did not incorporate the scientific quality of included studies when formulating conclusions (AMSTAR domain 8). This domain is facilitated by the use of GRADE, where explicit criteria are used for rating the quality of evidence, including study design, risk of bias, imprecision, inconsistency, indirectness, and magnitude of effect [14]. GRADE is now used by most new and updated Cochrane reviews. In line with Cochrane’s strict policy on conflicts of interest [22], all reviews included clear disclosures from the authors. However, about 40% of reviews failed to report on potential conflicts of interest in the primary studies included in the reviews, as required for the AMSTAR domain 11.

Among the completed reviews, only about a quarter (27%; *n* = 129) had review content assessed as up-to-date between 2013 to 31 July 2015 indicating that the most recent searches for new studies had been conducted during this period. Thus, the large majority of reviews had searches which were potentially out-of-date. Few studies have been conducted to inform decisions about how and when to update systematic reviews [23]. This is an evolving area and Cochrane’s approach of updating every review every two years has become unfeasible, and is currently undergoing a process of adaptation to develop clear and sensible guidance on updating of systematic reviews that considers whether the review addresses a current question, uses valid methods, and is well conducted; and whether there are new relevant methods, new studies, or new information on existing included studies [23]

Nutrition reviews, completed or in progress, covered a wide range of conditions and were widely distributed across 47 of the 53 CRGs (Fig. 3). Cochrane nutrition reviews reflect the current double nutrition burden, covering conditions associated with undernutrition and obesity. Reviews addressing the top causes of global YLLs [16] mainly addressed preterm birth, diabetes and ischaemic heart disease, and those targeting leading risk factors for DALYs [15] predominantly addressed childhood undernutrition and high body mass index.

### Paradigms of review topics

Although AMSTAR critically appraises the methodological quality of systematic reviews, it does not explicitly examine the perspective of the review question or its associated scope and complexity. We attempted to address this by categorising the completed nutrition reviews into the medical, lifestyle or socio-ecological paradigms.

Most review questions were formulated within a medical paradigm, with half of the reviews examining supplementation/supplement interventions, mostly with micronutrients. Micronutrient supplementation, using single or multiple nutrients, targets acute micronutrient deficiencies in high risk populations, and like other nutrition-specific interventions, mainly addresses the immediate causes of undernutrition. By contrast only one in five Cochrane nutrition reviews examined food-based interventions, which were mainly interventions to modify diets. Food-based interventions are generally more context-rich and complex than nutrient-based interventions, typically require behaviour change and have intended outcomes that are mostly not achievable in the short-term. These topics are usually located within a lifestyle paradigm. Fortification and supplementation programmes (e.g. vitamin A and zinc supplementation) are often prioritised over behaviour-change and health promotion-based approaches (e.g. exclusive breastfeeding and dietary diversity) as they are deemed more feasible [2]. An important reason for this may be that behaviour change is difficult to achieve without addressing underlying systemic and structural factors. It should, however, be kept in mind that in the absence of nutritional deficiency, effects of single nutrients and foods can be hard to demonstrate. Foods provide a complex mixture of nutrients and other components that may have synergistic, additive or antagonistic effects on health. Thus, in many instances, zooming in on a single dietary component runs the risk of ignoring the wider contexts and complex relationships between diet and health. This emphasises the importance of studying the effects of dietary patterns, a significantly neglected topic in the Cochrane nutrition reviews we assessed. Dietary patterns are defined as the quantities, proportions, variety, or combination of different foods, beverages, and nutrients (when available) in diets, and the frequency with which they are habitually consumed [24]. Evaluating dietary patterns can help identify links between the overall diet and its component beverages, foods and nutrients in relation to health outcomes, and thus reduces the multicollinearity among single foods and nutrients. Assessing dietary patterns can account for the intrinsic interactions between foods and nutrients that are relevant to effects on disease risk. It can also identify potential cumulative effects of single dietary components, and dietary exposure over time [24], in line with the lifestyle paradigm.

Factors unrelated to diet play an important role in the health impact of diets. Malnutrition is caused by the interaction between poor nutritional quality diets, unhealthy environments and health damaging behaviours. All three factors can be significantly shaped by underlying drivers, such as social disadvantage, income inequality, political instability and conflict, and some elements of globalization. It has been estimated that scaling up direct undernutrition interventions to 90% coverage rates will address only 20% of the stunting burden and that actions in other sectors (agriculture; health; education; social protection; water, sanitation, and hygiene) are vital to address the remaining 80% [2]. This complexity is encapsulated in the socio-ecological paradigm, which is currently under-represented in Cochrane nutrition reviews.

### Methodological considerations 

Only about 5% of nutrition reviews included study designs other than RCTs, likely because Cochrane reviews focus on questions related to effectiveness, where RCTs are considered the ‘gold standard.’ However, this may have the consequence of restricting the scope of review topics that can be considered [6, 25–27]. Some questions about effects in the field of nutrition are difficult to answer using a trial design due to long time horizons for outcomes of interest, ethical issues where there is potential for harm, high cost, lack of feasibility, and complexity. We believe that evidence from non-randomised and observational studies should be considered for inclusion in future Cochrane nutrition reviews, despite their known limitations, as they may provide the best available evidence to guide decision-making in some circumstances. For example, interventions for improving access to food in low- and middle-income countries and interventions to improve the implementation of healthy eating and obesity prevention policies or programmes within childcare settings. This is especially warranted in light of the availability of instruments that can be utilized to evaluate limitations of various designs and thereby reduce the likelihood of readers being misled by problems such as bias or confounding [28, 29]. Indeed the Cochrane Handbook for Systematic Reviews allows for the inclusion of studies other than RCTs [30]. Cochrane Methods Groups are actively developing methods for including non-randomized studies in reviews [31], assessing risk of bias for non-randomized studies [32], and for synthesising qualitative evidence and integrating it in reviews of intervention effects [29]. This methodological support network can assist by extending the range of study designs used in Cochrane nutrition reviews thereby expanding the number of important and challenging questions that can be addressed.

Currently, two thirds of nutrition reviews examine treatment questions for specific conditions (e.g. vitamin A for treating measles in children) with the rest addressing risk factor questions (e.g. altered dietary salt for preventing pre-eclampsia). Many of the treatment questions for undernutrition have been addressed, and we also know a great deal about the drivers and the types of interventions needed to address undernutrition [2, 33]. For other forms of malnutrition, such as overweight and obesity, the evidence base is more complex and fractured and many “what works” questions remain [2]. For all forms of malnutrition and particularly the co-existence of different forms of malnutrition, less is known about what combinations of interventions work best. For example, how to implement the right mix of nutrition actions in different contexts, equitably and affordably, at a scale that matches the size of the problem, and in ways that connect nutrition-specific and nutrition-sensitive interventions [33]; how to scale up coverage of proven nutrition-specific interventions and integrate nutrition actions into health system platforms [2]. We believe that future Cochrane nutrition evidence can contribute to addressing these knowledge gaps, thereby informing public health nutrition policies and programmes focussing on nutrition problems across all relevant sectors. Reviews of more complex issues can help to identify appropriate questions for more targeted systematic reviews and primary studies, prioritize topics for future research, map the nature of best available evidence (location, intervention, study methods, and study quality), and establish the existence, nature, and direction of reported impact, including intermediate and adverse impacts [34]. We plan on progressing this work in the future to inform activities to strengthen methods for preparing nutrition systematic reviews. For example, this will include nutrition-focused appraisals of systematic reviews in order to help address nutrition-specific issues in evidence synthesis, such as assessing baseline exposures to nutrients, nutrient status of participants, bioequivalence of nutrients, multiple and related biological functions of nutrients and time-scale, including the plausibility of a measurable effect over the duration of the studies [9].

Lavis and colleagues [35] have called for more innovation in the preparation of systematic reviews to improve their utility, and have highlighted the need for improved relevance and accessibility of reviews, while maintaining the rigour that is foundational to a systematic approach. Seminal work has been published in a series of six technical reports by the Agency for Healthcare Research and Quality [9], seeking to facilitate a better understanding of the challenges involved in preparing and using nutrition-related systematic reviews, and there are ongoing capacity-building initiatives for nutrition systematic reviews [36]. Cochrane can build on these advances to promote better nutrition evidence synthesis and improve the use of this evidence. This is in line with the objectives of the recently established Cochrane Nutrition Field, which are to increase the coverage, quality, and relevance of Cochrane nutrition reviews, increase the impact of Cochrane nutrition reviews across all stakeholders and contribute to strengthening methods for conducting nutrition systematic reviews. Activities will include consultative efforts to explore and delineate priorities for nutrition evidence synthesis. In achieving its objectives, Cochrane Nutrition plans to collaborate and engage with stakeholders within and outside of Cochrane, including other evidence synthesis initiatives.

## Conclusions

This analysis presents a comprehensive description of the scope and quality of Cochrane nutrition reviews, and identifies gaps for future activities to support actions to address the nutrition burden, in line with the current nutrition agenda and impetus. As the world seeks to accelerate and sustain recent nutrition gains, meet targets and reach places and people who have been left behind, a constant flow of new, good quality evidence is needed to fill knowledge gaps, deliver even greater impacts for existing resources, and to make the case for additional resources. Rigorous and relevant synthesised nutrition evidence, such as that from Cochrane systematic reviews, has a valuable role in informing nutrition decisions at local and international levels. It can also harness the political capital required from policymakers to reform policy and finance the scale up of interventions.
